# Syrup of Chloral

**Published:** 1881-11

**Authors:** 


### SYRUP OF CHLORAL.

It is a difficult matter to cover the acrid taste of chloral, but R. F. Fairthorne has found this difficulty overcome to a considerable extent in the formula here given:

℞ Chloral hyd. cryst. . . . . . . . . . ℈ iv.

Aqua menth. pip. . . . . . . fl ʒ iij.

Curacoa cordial . . . . . . . . fl ʒ iv.

Syrup acaciæ, q.s. ut ft . . . . . fl ℥ ij.

*American Journal of Pharmacy*

